# Potential Therapeutic Effects of Nitrate/Nitrite and Type 2 Diabetes Mellitus

**DOI:** 10.5812/ijem.9103

**Published:** 2013-04-01

**Authors:** Asghar Ghasemi, Saleh Zahediasl

**Affiliations:** 1 Endocrine Physiology Research Center, Research Institute for Endocrine Sciences, Shahid Beheshti University of Medical Sciences, Tehran, IR Iran

**Keywords:** Diabetes, Nitrate, Nitrite

Type 2 diabetes, and it is expected to rise to 439 million by 2030 (1). Currently although insulin and oral ant diabetic agents are used for treatment of diabetes, most of them usually have inadequate efficacy and adverse effects and therefore new strategies need to be examined (2). Type 2 diabetes is a complex metabolic disease and decreased beta-cell mass, beta-cell dysfunction, and increased insulin resistance have been reported to be important in its pathogenesis; however abnormal pulsatility of basal insulin secretion, loss of first-phase of insulin release, and increased glucagon secretion also contribute to the development of type 2 diabetes (3, 4). Nitric oxide (NO) is a simple ubiquitous molecule that plays important roles in almost every biological system (5). NO is synthesized from L-arginine by NO synthase (NOS) enzymes including endothelial (eNOS), neuronal (nNOS), inducible (iNOS), and mitochondrial (mtNOS) NOS. Approximately 90% of NO in the body is converted to nitrate, which is the main stable end-product of NO in vivo (5).

Inorganic nitrate (NO3-) and nitrite (NO2-) mostly considered as potentially harmful constituents in our food and drinking water (6, 7). According to WHO guidelines, upper limits for nitrate and nitrite are 50 mg/L and 3 mg/L respectively and the sum of the ratios of the concentration of each to its guideline value should not exceed 1 (8). The view that nitrate and nitrite are harmful is now being questioned after NOS-independent NO formation from nitrate and nitrite, was reported in 1994 (9); it seems that nitrate-nitrite-NO pathway is complementary or even a back-up system (mostly during hypoxia and low pH) to the classical L-arginine-NO synthase pathway (7). Recent reports show that nitrate and nitrite may have some therapeutic and nutritional implications (5, 7) including protecting against ischaemia-reperfusion injury in mice (10), decreasing blood pressure in humans (11, 12) and animals (13), reducing oxidative stress (7), improved mitochondrial efficiency due to reduced uncoupling in humans (14), and reducing oxygen consumption during exercise, which may improve physical capacity in disease with limited pulmonary function (7). It has been suggested that reduced NO availability is a central event in the pathogenesis of metabolic syndrome (15) and 10-week inorganic nitrate therapy reduces visceral fat accumulation, lowers serum triglycerides in eNOS deficient mice, and normalizes a disturbed glucose tolerance (16). These findings may have implications in prevention and treatment of type 2 diabetes (15) considering that long-term nitrate therapy also reduces weight gain (16, 17). In addition, L-arginine can evoke insulin release and it seems that NO may be physiologically involved in the release of insulin (18).

These potential therapeutic effects should however be considered in the context of reports on the harmful effects of nitrite/nitrate and L-arginine; induced NO formation may play a role in the destruction of the pancreatic beta cells during development of type 1 diabetes in mice (18). NO donors have been also shown to cause β cell dysfunction and damage through IL-1β (19). In fact, NO causes FFA-induced decreased insulin secretion in prediabetic rat islets (19); iNOS is involved in the development of muscle insulin resistance in diet-induced obesity by affecting PI 3-kinase and Akt in mice (20) and the iNOS mRNA was 20 times higher in diabetic rats (19). High levels of plasma nitrate increase arterial pressure (21) and nitrate may cause early onset of hypertension and increased incidence of diabetes (6). In addition nitrate may induce kidney dysfunction (17) and long-term nitrate therapy, at least in animal studies, may produce hypothyroidism (17). Cancer, particularly of the stomach, is another concern attributed to nitrate (5); nitrate acts as a precursor in the formation of N-nitroso compounds, which are carcinogens in animals (22); however no causative link between nitrite and nitrate exposure and cancer including stomach cancer has been documented in humans (5, 23).

In conclusion, there is a dichotomy of current scientific attitudes about the use of nitrate and nitrite; i.e. having potential new therapeutic application for human health or having potential human risks and further researches are needed in this field, especially to identify individuals that may benefit from nitrate/nitrite therapy. It seems that nitrate/nitrite therapy would be potentially beneficial, especially when derived from natural sources such as vegetables.
